# Editorial: New methods and approaches in toxicology of emerging environmental contaminants

**DOI:** 10.3389/ftox.2026.1930982

**Published:** 2026-07-28

**Authors:** Martin Ezechiáš, Michal Šíma, Jaroslav Semerád

**Affiliations:** 1 The Laboratory of Environmental Biotechnology, Institute of Microbiology of the Czech Academy of Sciences, Prague, Czechia; 2 Department of Toxicology and Molecular Epidemiology, Institute of Experimental Medicine of the Czech Academy of Sciences, Prague, Czechia; 3 Institute for Environmental Studies, Faculty of Science, Charles University, Praha, Czechia

**Keywords:** computational toxicology, effect-based monitoring, emerging environmental contaminants, environmental risk assessment, mechanistic toxicology, new approach methodologies (NAMs)

Emerging environmental contaminants represent an increasing challenge for toxicological research and risk assessment. The growing diversity of anthropogenic chemicals, novel materials, pharmaceuticals, endocrine disruptors, micro- and nanoplastics, and other types of nanoparticles, together with the complexity of realistic exposure scenarios, has substantially exceeded the capacity of traditional toxicological approaches based primarily on individual compounds and apical endpoints. In addition, chronic low-dose exposures and interactions among multiple stressors further complicate the interpretation of adverse effects and their implications for human and environmental health. The widespread occurrence of these contaminants and the diversity of exposure scenarios, including occupational exposure in some populations, further highlight the need for new methods and approaches for hazard identification and risk assessment.

Recent advances in cell-based systems, omics technologies, computational tools, machine learning, and effect-based monitoring have created unique opportunities to improve our understanding of toxicity mechanisms and to develop more predictive and biologically relevant assessment strategies. Consequently, toxicology is increasingly moving from descriptive observations toward mechanistic, human-relevant, and environmentally realistic approaches. New approach methodologies (NAMs), systems toxicology, passive sampling, and artificial intelligence are increasingly complementing conventional methods and provide new perspectives for hazard identification and risk assessment.

This Research Topic comprises thirteen contributions, including original research articles, reviews, a mini-review, and a brief research report, highlighting recent advances in mechanistic toxicology, computational methods, effect-based approaches, and environmentally relevant risk assessment of emerging contaminants.

Several contributions in this Research Topic illustrate the growing importance of mechanistic toxicology and human-relevant models. Iulini et al. employed human peripheral blood mononuclear cells and dendritic cell models to investigate the immunotoxicity of per- and polyfluoroalkyl substances (PFAS). Their findings demonstrated that immunotoxic potential cannot be explained solely by carbon chain length and emphasized the need for compound-specific assessment strategies. Similarly, Muhammad et al. reviewed the contribution of micro- and nanoplastics to Parkinson’s disease and highlighted the advantages of stem cell-derived models for investigating neuroinflammation and neurodegeneration. These systems may improve human relevance and allow detailed investigation of disease-associated pathways, while complementing existing animal models.

Mechanistic insights were further expanded by Pan et al., who demonstrated that perinatal exposure to bisphenol S induces ferroptosis through disturbances in hepatic lipid metabolism in offspring mice. These findings further support the need for careful toxicological evaluation of replacement chemicals and their potential long-term effects. At the organismal level, Pacchini et al. investigated the effects of chronic PFAS exposure in European chub and revealed coordinated responses extending from gene expression and antioxidant enzymes to tissue morphology. Their work illustrates how persistent environmental contamination can trigger adaptive responses across multiple levels of biological organization, from genes to organs.

Advances in computational toxicology and systems-based approaches are represented by the study of Li et al., who integrated network toxicology, machine learning, molecular docking, molecular dynamics simulations, and experimental validation to identify DEHP-associated targets and molecular signatures relevant to glioma biology. By combining several computational approaches with *in vitro* confirmation, the study provides an example of how artificial intelligence and systems toxicology can contribute to predictive toxicology.

Another important theme emerging from this Research Topic concerns the complexity of environmental exposures and the increasing importance of effect-based approaches. Ambroz et al. investigated oxidative stress and inflammatory biomarkers in mothers and newborns from environmentally distinct regions and demonstrated differential associations between pollutant exposure and oxidative damage. Their work highlights the importance of considering developmental susceptibility and the complexity of exposure-response relationships.

Effect-based monitoring was further addressed by Ezechiáš et al., who combined passive sampling with cell-based bioassays to evaluate the efficiency of drinking water treatment plants. Persistent *CYP1A* induction indicated residual bioactivity not apparent from conventional chemical analyses and emphasized the value of effect-based monitoring for assessing drinking water treatment efficiency. Fu et al. investigated combined exposure of zebrafish to cadmium and β-cypermethrin and demonstrated that co-exposure induced stronger oxidative stress responses and more severe histopathological alterations than individual compounds, emphasizing the ecological relevance of mixture toxicity. Complementing these studies, Alblooshi reviewed the health risks associated with perfumes and cosmetic products and discussed concerns related to cumulative exposure to endocrine disruptors, volatile organic compounds, and heavy metals. Together, these contributions illustrate the need to move beyond the traditional one chemical-one effect paradigm and adopt approaches capable of capturing realistic exposure scenarios.

Several contributions focused on improving methodologies for environmental risk assessment. Nugnes et al. proposed a comprehensive framework for standardized ecotoxicological assessment of titanium dioxide-based sunscreen leachates under realistic freshwater and marine conditions. Their work demonstrated the value of combining multi-species testing with realistic exposure scenarios. Machado et al. evaluated the ecotoxicity of montmorillonite-based essential oil formulations and demonstrated that bio-based products and natural carriers require rigorous ecotoxicological evaluation despite their natural origin. These results suggest that products perceived as sustainable alternatives should also undergo comprehensive safety assessment.

Environmental dimensions of toxicology were further addressed by Gil et al., who examined antifungal contamination from a One Health perspective and proposed predicted no-effect concentrations to support wastewater management and agricultural applications. Their study further illustrates the close links between environmental contamination, antimicrobial resistance, and public health, emphasizing the need for integrated management strategies.

An emerging issue highlighted in this Research Topic concerns the omnipresence of plastic particles and their potential influence on experimental systems. Lundqvist et al. demonstrated that routine laboratory procedures involving plastic tubes and ultrasound treatment can generate considerable amounts of nanoplastic particles that may influence experimental outcomes. These observations draw attention to hidden sources of contamination and raise important questions regarding the interpretation and reproducibility of studies involving nano- and microplastics.

Several challenges remain in advancing next-generation toxicology of emerging contaminants. These include the interpretation of mixture effects, the translation of mechanistic findings into regulatory frameworks, the validation and standardization of novel methodologies, and the integration of large experimental and computational datasets. Although many emerging approaches have demonstrated considerable promise, their broader implementation still requires robust performance standards, reproducibility, and demonstration of fitness for purpose.

Future progress in the toxicology of emerging contaminants will likely depend on the continued integration of human-relevant models, omics technologies, machine learning, effect-based monitoring, and systems toxicology, together with the broader adoption of One Health principles emphasizing the interconnected nature of environmental quality, ecosystem integrity, and human health. Overall, the contributions assembled in this Research Topic reflect the diversity of contemporary toxicological research and demonstrate how experimental, computational, effect-based, and environmentally realistic approaches are increasingly being integrated into the assessment of emerging environmental contaminants. Collectively, these studies illustrate the ongoing evolution of toxicology toward more predictive and biologically relevant frameworks for the assessment of emerging environmental contaminants.

